# Comparative Analysis of Chrysanthemum Stunt Viroid Accumulation and Movement in Two Chrysanthemum (*Chrysanthemum morifolium*) Cultivars with Differential Susceptibility to the Viroid Infection

**DOI:** 10.3389/fpls.2017.01940

**Published:** 2017-11-10

**Authors:** Tomoyuki Nabeshima, Motoaki Doi, Munetaka Hosokawa

**Affiliations:** Laboratory of Vegetable and Ornamental Horticulture, Graduate School of Agriculture, Kyoto University, Kyoto, Japan

**Keywords:** viroid resistance, chrysanthemum, Chrysanthemum stunt viroid, grafting, agrobacterium, Chrysanthemum chlorotic mottle viroid, RNA trafficking

## Abstract

Chrysanthemum stunt viroid (CSVd) was inoculated into two chrysanthemum (*Chrysanthemum morifolium*) cultivars, the CSVd-susceptible cultivar Piato and the CSVd-resistant cultivar Mari Kazaguruma. For CSVd inoculation, grafting and *Agrobacterium*-mediated inoculation were used. In grafting experiments, CSVd was detectable in Mari Kazaguruma after grafting onto infected Piato, but after removal of infected rootstocks, CSVd could not be detected in the uppermost leaves. In agroinfection experiments, CSVd systemic infection was observed in Piato but not in Mari Kazaguruma. However, agro-inoculated leaves of Mari Kazaguruma accumulated circular CSVd RNA to levels equivalent to those in Piato at 7 days post-inoculation. *In situ* detection of CSVd in inoculated leaves revealed that CSVd was absent in phloem of Mari Kazaguruma, while CSVd strongly localized to this site in Piato. We hypothesize that CSVd resistance in Mari Kazaguruma relates not to CSVd replication but to CSVd movement in leaves.

## Introduction

Viroids are small, circular, highly structured RNAs of approximately 250–400 nt without a protein-coding region. They are the smallest infectious agents in plants and cause various symptoms (Diener, 2003). They have been found to infect various economically important crops, including vegetables, field crops, fruits or palm trees and ornamentals, and in sometimes severe symptoms cause significant reduction in productions (Hadidi et al., 2017; Hammond, 2017; Rodriguez et al., 2017; Verhoeven et al., 2017). In addition to direct effect on production, three species of viroids are listed for quarantine actions by EPPO and it can be said that viroids have significant impact on international trades of plant materials also (Barba and Hadidi, 2017). Thus far, 32 species of viroids have been identified, and they are grouped into two families, *Pospiviroidae* and *Avsunviroidae* (Di Serio et al., 2014). The members of *Pospiviroidae* have a central conserved region in their genome (Keese and Symons, 1985) and adopt a rod-shaped secondary structure (Sänger et al., 1976). The biosynthesis of *Pospiviroidae* is achieved *via* asymmetric rolling-circle replication in a host nucleus (Flores et al., 2009). In this system, monomeric circular (mc) forms are templates for multimeric, complementary, antisense sequences, followed by secondary RNA–RNA transcription to multimeric sense sequences. These multimeric sense sequences are then cleaved into unit lengths and circularized to produce mc forms. RNA polymerase II (Mühlbach and Sänger, 1979), class III RNase (Gas et al., 2007), and DNA ligase I (Nohales et al., 2012) have been implicated as host factors for their replication. Members of the family *Avsunviroidae* do not have a central conserved region but have highly branched structures with a self-cleaving ribozyme (Navarro and Flores, 1997; Flores et al., 2000). They replicate and accumulate in the chloroplast and their biosynthetic pathway is symmetric, where multimeric, antisense, linear RNAs are self-cleaved into monomeric linear RNAs and circularized to become templates for producing multimeric sense sequences that in turn are cleaved and circulized (Branch and Robertson, 1984; Flores et al., 2000).

Chrysanthemum (*Chrysanthemum morifolium*; 2n = 6x = 54) is an economically important ornamental plant cultivated throughout the world. *Chrysanthemum stunt viroid* (CSVd) and *Chrysanthemum chlorotic mottle viroid* (CChMVd) are known to infect chrysanthemums (Cho et al., 2013). CChMVd is a member of the *Avsunviroidae* family and has been identified as the causal agent of chrysanthemum chlorotic mottle disease by Navarro and Flores (1997). Such a disease causes yellow-green mottling, chlorosis, and stunting in host plants (Dimock and Geissinger, 1969; Dimock et al., 1971). Another viroid that infects chrysanthemums, CSVd, is one of the most serious pathogens in chrysanthemum production. CSVd is a member of the *Pospiviroidae* family and has been identified as the causal agent of chrysanthemum stunt disease (Diener and Lawson, 1973). CSVd shares 69% sequence identity with and can form a secondary structure similar to that of *Potato spindle tuber viroid* (PSTVd), the type species of the family *Pospiviroidae* (Haseloff and Symons, 1981). To date, various strains of CSVd have been sequenced, and their lengths range between 354 nt and 356 nt (Haseloff and Symons, 1981; Yoon and Palukaitis, 2013). Symptoms caused by this pathogen, include stunting, leaf spot, poor rooting, flower size reduction, and disturbance of photoperiodic response of flowering initiation (Dimock, 1947; Brierley and Smith, 1951; Horst et al., 1977; Hosokawa et al., 2004b). Strategies for managing chrysanthemum stunt disease include establishment of viroid-free plant production methods (Bachelier et al., 1976; Barba et al., 2003; Hosokawa et al., 2004a), creating resistant lines by transgenic approaches (Ogawa et al., 2005; Jo et al., 2015), and development of anti-viroid agent (Iraklis et al., 2016). Searching for natural resistance and its application in future breeding is one of the most important goals in chrysanthemum protection. In our previous study, we evaluated 85 commercial cultivars for CSVd resistance and found four CSVd-resistant chrysanthemum cultivars that showed slow-accumulation of CSVd after grafting onto infected rootstocks (Nabeshima et al., 2012). In the subsequent study, we further investigated 199 cultivars for CSVd resistance and chose four resistant cultivars (Nabeshima et al., 2014). In these resistant cultivars, CSVd could be detected after grafting onto infected rootstocks, but the titers in upper leaves were less than 1.0 × 10^-3^ to 1.0 × 10^-6^ of those in susceptible cultivars (Nabeshima et al., 2012, 2014). However, mechanisms underlying these resistance phenotypes have not been elucidated. Because some CSVd-resistant traits were reported to be inheritable (Omori et al., 2009; Matsushita et al., 2012), characterizing these resistant traits is important for future breeding to select ideal parental plants and combine multiple resistant traits for strong and durable resistance.

In this study, we focused on phenotyping the cultivar Mari Kazaguruma. This cultivar showed no visible symptom after grafting-mediated CSVd inoculation, and CSVd titers in upper leaves were almost 1/10^-6^ of those in susceptible cultivars (Nabeshima et al., 2012). Successful proliferation of viroids of the *Pospiviroidae* family in host plants includes the following steps: nuclear import, replication in the nucleus, nuclear export, cell-to-cell movement, and long-distance movement though the phloem (Tabler and Tsagris, 2004; Flores et al., 2005; Ding, 2009; Palukaitis, 2014). In an attempt to clarify which steps were responsible for CSVd slow-accumulation phenotype in Mari Kazaguruma, we did a comparative analysis of the CSVd infection in Mari Kazaguruma and the highly susceptible cultivar Piato.

## Materials and Methods

### Plant Materials

*Chrysanthemum morifolium* cvs. Piato and Mari Kazaguruma were vegetatively propagated by cutting conducted *in vitro*. Piato is a cultivar susceptible to CSVd and CChMVd (Hosokawa et al., 2004a, 2005). CSVd and CChMVd-free Piato was obtained by leaf primordia-free shoot apical meristem culture method (Hosokawa et al., 2004a, 2005), and viroid-free status has been maintained *in vitro*. Mari Kazaguruma plants were obtained from a chrysanthemum nursery (Seiko-en Co., Fuchu, Japan). CSVd and CChMVd-free status of plants was checked routinely by Reverse transcription (RT)- polymerase chain reaction (PCR) as described below. Plants were grown on Murashige and Skoog (MS) medium (Murashige and Skoog, 1962) with 10% strength ammonium nitrate. Incubation conditions were 25 ± 3°C, 3500 lx, and a 16 h photoperiod provided by cool white fluorescent light tubes. P4 was a clonally propagated line of Piato, which has been infected by CSVd and CChMVd for more than 10 years and retains high and stable CSVd and CChMVd titers. At least four strains of CSVd (GenBank: AB689034.1, AB689033.1, AB689032.1, and AB689031.1) were naturally infected onto P4 (Nabeshima et al., 2012). P4 clonally propagated lines were used as rootstocks in grafting experiments and positive controls in RT-PCR analysis.

### CSVd Inoculation by Grafting

A shoot tip about 10 mm in length was used as a scion. All expanded leaves were removed before grafting. Scions were grafted onto root-less P4 cuttings with two expanded leaves. The graft position was gently held by sterilized wool string (about 2 mm diameter). Plantlets were grown in glass test tubes containing MS medium. Incubation conditions were as described above. The upper part of grafted scions with four expanded leaves was cut from grafted plants 40 days after grafting and rooted in new MS medium. Cuttings were again made after another 40 days after the first cutting (80 days after grafting), and they were continuously grown in MS medium until 140 days after grafting. During the growth period, CSVd titers in the uppermost expanded leaves were examined as described below. Twelve scions for each cultivar were used for grafting, but two plants of Piato and four plants of Mari Kazaguruma were exposed to fungal contamination during their growth, and they were excluded from the experiment after contamination was observed. After 140 days of grafting, three plants of both cultivars were cut for obtaining new scions with four expanded leaves and then rooted in new MS medium. When they had grown to have 10 expanded leaves, roots and the uppermost, middle, and bottom leaves were collected for RNA extraction and used for detection of CSVd and CChMVd by real-time RT-PCR as described below.

### Micro-Tissue (MT) Direct Real-Time RT-PCR

For time-course analysis of CSVd titers in grafted scions, micro-tissue (MT) direct real-time RT-PCR (Hosokawa et al., 2006; Nabeshima et al., 2014) was performed. A syringe needle (0.50 × 25 mm; Terumo, Tokyo, Japan) was used to pierce expanded leaves once at random positions, and plant sap on the syringe needle was used as source of template by dipping into the PCR reaction mixture. The reaction mixture comprised 6.5 μl One Step SYBR^®^ RT-PCR Buffer 4 (TaKaRa Bio Inc., Tokyo, Japan), 0.6 μl TaKaRa Ex Taq^®^ HS Mix (TaKaRa Bio Inc.), 0.2 μl PrimeScript^®^ PLUS RTase Mix (TaKaRa Bio Inc.), 0.2 μl Rox Reference Dye (TaKaRa Bio Inc.), 0.4 μM of each of forward [CSVd-NF: 5′-CCAATCTTCTTTAGCACCGG-3′; Hosokawa et al. (2004a)] and reverse [CSVd-NR: 5′-AGTGGGGTCCTAAGCCCCAA-3′; Hosokawa et al. (2004a)] primers, and was adjusted to 10 μl with sterilized distilled water. The reaction was performed with real-time RT-PCR (ABI PRISM^®^ 7900HT, Applied Biosystems, Foster City, CA, United States) and conducted with the following reverse transcription and cycling parameters: 5 min at 42°C, 35 cycles of 30 s at 95°C, and 30 s at 60°C. Efficient and specific amplification of target products without primer dimers was checked by dissociation curves. Four replicates in each leaf were conducted and the data shown are means. All data were standardized using the concentration of CSVd in expanded leaves of P133, another CSVd-infecting Piato line, as previously described (Nabeshima et al., 2014).

### Vector Construction

**FIGURE 1:**
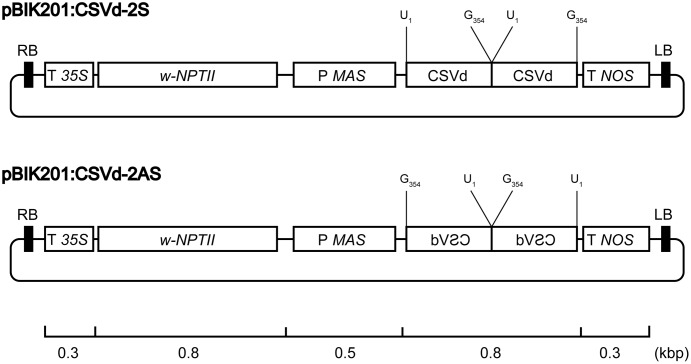
Physical maps of pBIK201:CSVd-2S and pBIK201:CSVd-2AS. RB: right border of T-DNA region, LB: left border of T-DNA region, T *35S*: *Cauliflower mosaic virus 35S* terminator, *NPTII*: *neomycin phosphotransferase II*, P *MAS*: *mannopine synthase* promotor, T *NOS*: *nopaline synthase* terminator.

Construction of binary vectors containing sense or antisense dimeric CSVd (GenBank: AB689034.1) in the *Xba*I-*Sac*I sites of pBIK201i:GUS (Shinoyama, unpublished), designated as pBIK201:CSVd-2S and pBIK201:CSVd-2AS, were previously described (Nabeshima et al., 2016). In pBIK201:CSVd-2S and pBIK201:CSVd-2AS, sense or antisense dimeric head-to-tail CSVd sequences are driven under regulation of the *mannopine synthase* promoter and the *nopaline synthase* terminator (**Figure 1**).

### RNA Extraction, Purification, and Detection of CSVd by RT-PCR

Total RNA was extracted from expanded leaves using Seprasol RNA 1 Super G (Nacalai Tesque, Kyoto, Japan) according to the manufacturer’s instructions. Extracted RNAs were purified once using a commercial agent [High-Salt Solution for Precipitation (Plant), TaKaRa Bio Inc.] according to the manufacturer’s instructions. RT was performed in a 5 μL volume containing 25 units of ReverTra Ace (Toyobo, Osaka, Japan), 0.5 μL RT buffer, 1 mM dNTPs (10 mM), 10 units of RNase inhibitor (Toyobo), and 1 μM reverse primer CSVd-R [5′-AGGATTACTCCTGTCTCGCA-3′; Hosokawa et al., 2004b]. Total RNA was added to the reaction mixture to a final concentration of 10–20 ng⋅μL^-1^, and then, this mixture was incubated at 42°C for 30 min and 99°C for 5 min. One μL of RT product was added to 9 μL of the PCR mixture to obtain a final volume of 10 μL. RT-PCR was performed in a 10 μL mixture containing 0.25 units of Blend Taq polymerase (Toyobo), reaction buffer for the Blend Taq polymerase, 0.2 mM dNTPs, and 0.2 μM forward and reverse primers. One cycle of 3 min at 98°C, 35 cycles of 30 s at 98°C, 10 s at 58°C, and 30 s at 74°C, and one cycle of 5 min at 74°C were performed. The primers CSVd-R and CSVd-F (5′-CAACTGAAGCTTCAACGCCTT-3′; Hosokawa et al., 2004b) were used. The RT-PCR products were separated by electrophoresis on a 1.0% agarose gel and visualized by ethidium bromide staining.

### Real-Time RT-PCR

Relative quantification of the CSVd and CChMVd titer was performed with real-time RT-PCR (ABI PRISM^®^ 7900HT). The chrysanthemum *Actin* gene (Narumi et al., 2005) was used as the internal standard. Reverse transcription was performed as described above. CSVd-NR and CChMVd-269R (5′-CCGAGGAGAATATCCAACGA-3′) were used for CSVd and CChMVd reverse transcription, respectively. The RT product was diluted 5 times by RNase-free distilled water, and then, two microliters were used for the real-time RT-PCR. The real-time RT-PCR mixture, composed of 10 μl SYBR Premix Ex Taq (TaKaRa Bio Inc.), 0.4 μl Rox reference dye (TaKaRa Bio Inc.), and 0.8 μM forward and reverse primers, was adjusted to 18 μl with sterile distilled water. Primers used were CSVd-NR and CSVd-NF for CSVd, CChMVd-269R (5′-CCGAGGAGAATATCCAACGA-3′) and CChMVd-28F (5′-ATCCATGACAGGATCGAAAC-3′) for CChMVd, and Actin F (5′-ACATGCTATCTTGCGTTTGG-3′; Narumi et al., 2005) and Actin R (5′-CTCTCACAATTTCCCGTTCA-3′; Narumi et al., 2005) for *Actin*. Forty cycles of 15 s at 95°C and 15 s at 60°C were performed. Efficient and specific amplification of target products without primer dimers was checked by dissociation curves. Data are shown as CSVd/*Actin* ratios.

### Agroinfection

pBIK201:CSVd-2S and pBIK201:CSVd-2AS were introduced into *Agrobacterium tumefaciens* strain EHA105 by the electroporation method. Transgenic agrobacterium was cultured overnight in liquid LB medium containing 50 ppm kanamycin at 28°C. Cultures were centrifuged (2500 *g*, 10 min) and then suspended in infiltration buffer [(10 mM MES pH 5.6, 10 mM MgCl_2_, and 200 μM acetosyringone (Sigma-Aldrich, St. Louis, MO, United States)] to an optical density of 2.0 at 600 nm. The resultant inocula were incubated at room temperature for at least 2 h and then used for infection. The fifth youngest expanded leaf was infiltrated with 10 μl of inoculum using a 1 mL syringe (SS01-T, Terumo) without needle. Plants infiltrated by infiltration buffer without agrobacterium were prepared as negative controls. For the analysis of intra-leaf CSVd movement, a syringe needle (25G × 1 inch, Terumo) was used for inoculation. In this case, a syringe needle was dipped in an agrobacterium culture and then used for piercing a leaf.

### Northern Blot Analysis

Total RNA was separated by electrophoresis in 5.0% polyacrylamide gels containing 0.5X TBE buffer and 8 M urea and transferred to Hybond-N++ (GE Healthcare Japan, Tokyo, Japan). Hybridization was performed overnight at 65°C in DIG easy Hyb (Roche Diagnostics, Basel, Switzerland) buffer. DIG-labeled RNA probes were synthesized by T7 RNA polymerase and *in vitro* transcription using a DIG RNA Labeling Kit (Roche Diagnostics). pTAC-1 (BioDynamics Laboratory Inc. Tokyo, Japan) plasmid harboring full-length, monomeric CSVd (Nabeshima et al., 2012) was linearized by *Xba*I and used as the template for probe synthesis. After hybridization, the membrane was washed twice in low stringency buffer (2.0% SSC and 0.1% SDS) for 5 min at room temperature and then washed twice in high stringency buffer (0.1% SSC and 0.1% SDS) for 15 min at 65°C. Detection of CSVd signals was conducted using Anti-DIG-AP Fab Fragments (Roche Diagnostics) and CDP-Star detection reagent (GE Healthcare Japan), and images were obtained by an image analyzer (LAS-3000 mini, FUJIFILM, Tokyo, Japan). For the quantification of circular RNA accumulation, band intensities corresponding to CSVd mc forms were measured using the gel analysis function of ImageJ 1.49P software. The same RNA dilutions used for northern blotting were separated in 1.0% agarose gels containing 4.9% formaldehyde (v/v) and 1X MOPS buffer and stained by ethidium bromide. Band intensities corresponding to 26S rRNA were analyzed to standardize concentrations of each RNA sample.

### Tissue Blot Analysis

Plants inoculated with pBIK201:CSVd-2S were used for tissue blot analysis at 20 and 25 days post inoculation (dpi) to examine CSVd distribution in above- and below-ground tissues, respectively. Stem and petioles of plants inoculated with pBIK201:CSVd-2S were cut into segments in lengths of approximately 7 mm by a razor blade, and each of their basal cut ends were pressed onto a nylon membrane. Blots were arranged on the membrane to indicate where they were originally positioned in the shoots. Root sap of inoculated plants were blotted on the membrane by pressing using a plastic roller. Hybridization and signal detection were performed as described above.

### *In Situ* Hybridization

*In situ* hybridization was performed as previously described (Nabeshima et al., 2012). Leaf samples were fixed with FAA solution [3.7% paraformaldehyde (w/v), 5% acetic acid (v/v) and 50% ethanol (v/v)] at 4°C overnight. Leaves infiltrated by EHA105 harboring pBIK201:CSVd-2S were collected at 14 dpi. Leaves inoculated with EHA105 harboring pBIK201:CSVd-2S by a needle were collected at 5 dpi. The fixed tissues were dehydrated and embedded in paraffin (Paraplast Plus, Sigma–Aldrich). The tissues were cut into 10-μm sections and dried overnight. Hybridization was performed overnight at 55°C using full-length CSVd antisense DIG-labeled RNA probes. Detection of signals was performed using anti-DIG alkaline phosphatase conjugate containing 0.1% BSA and NBT/BCIP (Roche Diagnostics) at 37°C.

## Results

### CSVd Inoculation by Grafting

**FIGURE 2:**
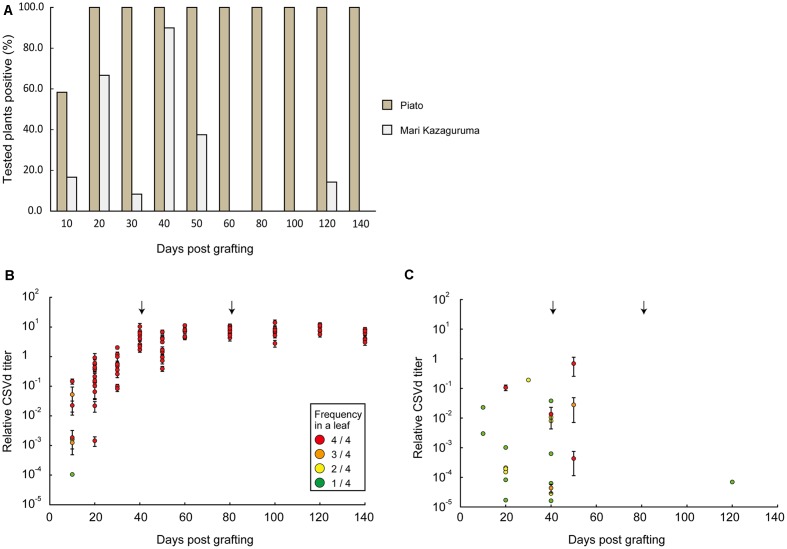
Time course analysis of CSVd infection in the uppermost leaves of Piato and Mari Kazaguruma grafted onto an infected Piato rootstock. **(A)** CSVd detection ratios in Piato and Mari Kazaguruma. CSVd detection was conducted by a Micro-tissue-direct real-time RT-PCR method. Four spots for each leaf were analyzed to determine CSVd titer, and when titers in all four spots were below detection limit, the plant was classified as “negative.” The other plants were classified as “positive,” regardless of the frequency of CSVd detection. **(B)** CSVd titers in CSVd-inoculated Piato. Each plot represents the mean of CSVd titers of four tested spots in a leaf of a single plant, standardized by the CSVd titer value of P133, a CSVd-infected line of Piato. The color of plots represents the number of CSVd-positive spots among four tested spots, as shown in the box. Bars indicate standard errors. Arrows indicate the days when cutting was conducted. **(C)** CSVd titers in CSVd-inoculated Mari Kazaguruma.

**FIGURE 3:**
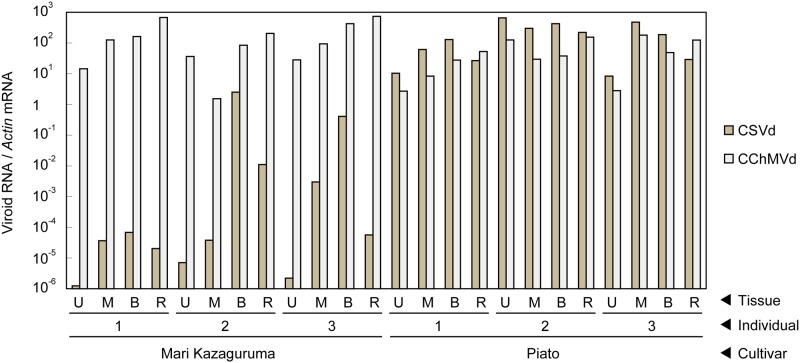
Vertical distribution of CSVd and CChMVd in Piato and Mari Kazaguruma. The titers of CSVd and CChMVd were determined by real-time RT-PCR. Data was standardized by expression level of *Actin* mRNA. Data was shown as means of three biological replications. Bars indicate standard errors.

Piato and Mari Kazaguruma plants were inoculated with CSVd and CChMVd by grafting onto infected Piato. First, we examined time-course changes of CSVd titers in the uppermost leaves. In Piato, CSVd was detected in all tested leaves at 20 days post grafting (dpg) and the titers reached a plateau at 40 dpg (**Figures 2A,B**). Although CSVd titers in the uppermost leaves were slightly decreased at 50 dpg (10 days after rootstock removal), titers reached levels almost identical to those just before cutting, and this level was maintained until the end of the experiment. CSVd titers were lower in Mari Kazaguruma than in Piato throughout the experimental period (**Figure 2C**). Some of the CSVd-detected Mari Kazaguruma plants accumulated CSVd to about 1–10th to 1–100th the levels in Piato until 50 dpg; however, later, CSVd decreased to levels of under detectable limit in any plant at any time point, except for a very faint titer in one plant at 120 dpg (**Figure 2C**). Then, three randomly selected Piato plants and three Mari Kazaguruma plants that showed CSVd accumulation at 50 dpg (**Figure 2C**) were chosen for further analysis. Scions with three expanded leaves were obtained from these plants at 120 dpg and were rooted in new media. When these plants grew to have 10 expanded leaves, roots and the uppermost, middle, and bottom leaves were assayed by real-time RT-PCR to determine CSVd and CChMVd titer. In Piato, CSVd and CChMVd accumulation was almost identical among different tissues and their viroid RNA/*Actin* mRNA ratios ranged from about 1.0 to 1.0 × 10^3^ (**Figure 3**). In Mari Kazaguruma plants, CChMVd titers in tested tissues were almost identical or even higher than in Piato. As for CSVd, we detected the lowest titers in the uppermost leaves, the highest titers in the bottom leaves, and intermediate titers in middle leaves and roots (**Figure 3**). Because the uppermost leaves developed after removal of infected rootstocks, it was considered that CSVd achieved systemic infection in Mari Kazaguruma, but the efficiency was quite low.

### CSVd Systemic Infection from a Single Leaf

**FIGURE 4:**
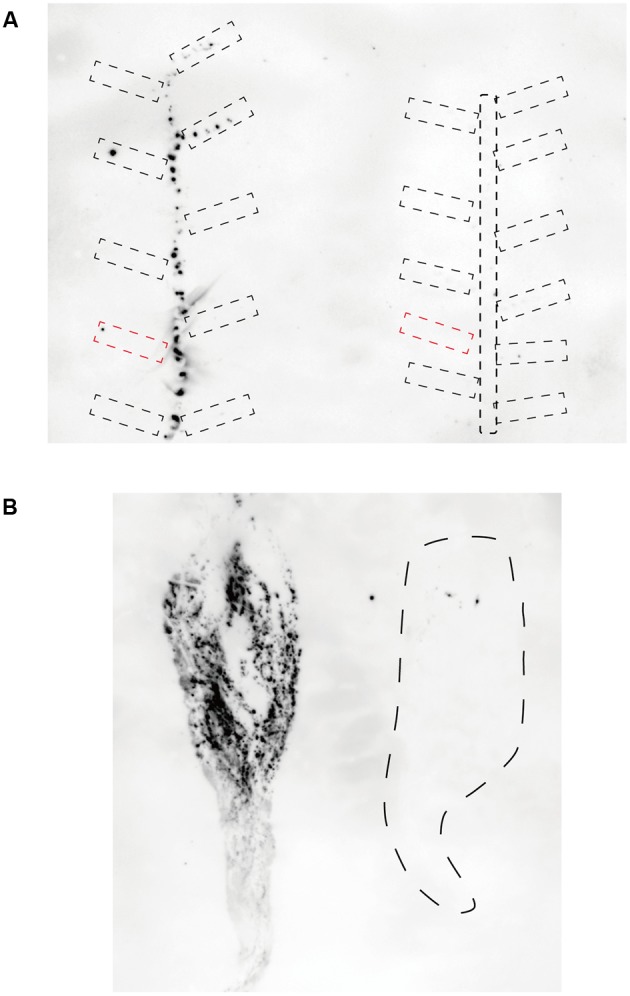
Detection of CSVd in Piato and Mari Kazaguruma by tissue blotting. Stem and petioles were cut into 7 mm segments, and their cut ends were blotted onto nylon membranes **(A)**. Root sap was blotted onto nylon membrane by pressure using a roller **(B)**. Hybridization was performed at 65°C using DIG-labeled CSVd full-length antisense probe. **(A)** CSVd infection in above-ground tissue of Piato (left) and Mari Kazaguruma (right) at 20 dpi. Broken circles indicate petioles. Note that petioles of inoculated leaf are represented by red, dashed boxes. **(B)** CSVd infection in underground tissues of Piato (left) and Mari Kazaguruma (right) at 25 dpi. Dashed line indicates the boundary of the tissue-blotted area.

To investigate CSVd proliferation in different cultivars, we adopted an *Agrobacterium*-mediated CSVd inoculation method. In both cultivars, we did not detect CSVd from non-inoculated, systemic leaves of three mock-inoculated plants by RT-PCR. Consistent with our previous report (Nabeshima et al., 2016), CSVd systemic infection was determined in upper, non-inoculated leaves of Piato at 20 dpi in pBIK201:CSVd-2S-inoculated (8 plants were used) and pBIK201:CSVd-2AS-inoculated (5 plants were used) plants. When pBIK201:CSVd-2S-inoculated leaves were detached after inoculation, CSVd could be detected in upper leaves at 25 dpi when inoculated leaves were detached at 6 dpi, but it could not be detected in upper leaves at 25 dpi when inoculated leaves were detached at 2 dpi (**Table 1**). Thus, CSVd translocation from inoculated leaves was considered to start 3–5 days after inoculation in Piato. In Mari Kazaguruma, CSVd could not be detected at either 20 or 25 dpi in 8 of pBIK201:CSVd-2S-inoculated plants and 5 of pBIK201:CSVd-2AS-inoculated plants. Tissue blotting experiments further provided contrasting pictures of CSVd systemic translocation in these two cultivars. At 20 dpi, positive strands of CSVd RNA could be detected in the stem, petioles of upper leaves, and roots in Piato (**Figure 4A**, left). In tomato (*Solanum lycopersicum*), PSTVd translocation from an inoculated leaf followed the distribution pattern of photoassimilates, and it did not move toward a source leaf (Palukaitis, 1987; Zhu et al., 2001). CSVd was absent in petioles of two leaves below and three leaves above the inoculated position, probably due to the fact that they were mature and had source leaf characteristics during the experimental period. In Mari Kazaguruma, CSVd could not be detected in either petioles of the upper leaves or in the stem (**Figure 4A**, right). At 25 dpi, roots of Piato accumulated high amounts of CSVd RNA, suggesting that roots were strong sinks for CSVd. However, in Mari Kazaguruma roots, CSVd signals were scarce (**Figure 4B**). From these results, it was considered that mature leaves of Mari Kazaguruma were not strong sources for CSVd systemic translocation.

**Table 1:** Systemic infection of CSVd in agrobacterium-infiltrated Piato following removal of infiltrated leaves.

Inoculum	Removal	Number of	Circular	Infection in
		plants used	CSVd^a^	systemic
				leaves^b^
Mock	-	3	0	0
pBIK201:CSVd-2S	2 dpi.	4	4	0
	4 dpi.	4	4	2
	6 dpi.	4	4	4

^a^*Number of plants which accumulated detectable amount of monomeric circular CSVd in inoculated leaves at the time of removal. ^b^CSVd infection in uninoculated upper leaves were determined by RT-PCR at 20 dpi in Piato and 25 dpi in Mari Kazaguruma*.

### Accumulation of Circular CSVd RNA in Inoculated Leaves

**FIGURE 5:**
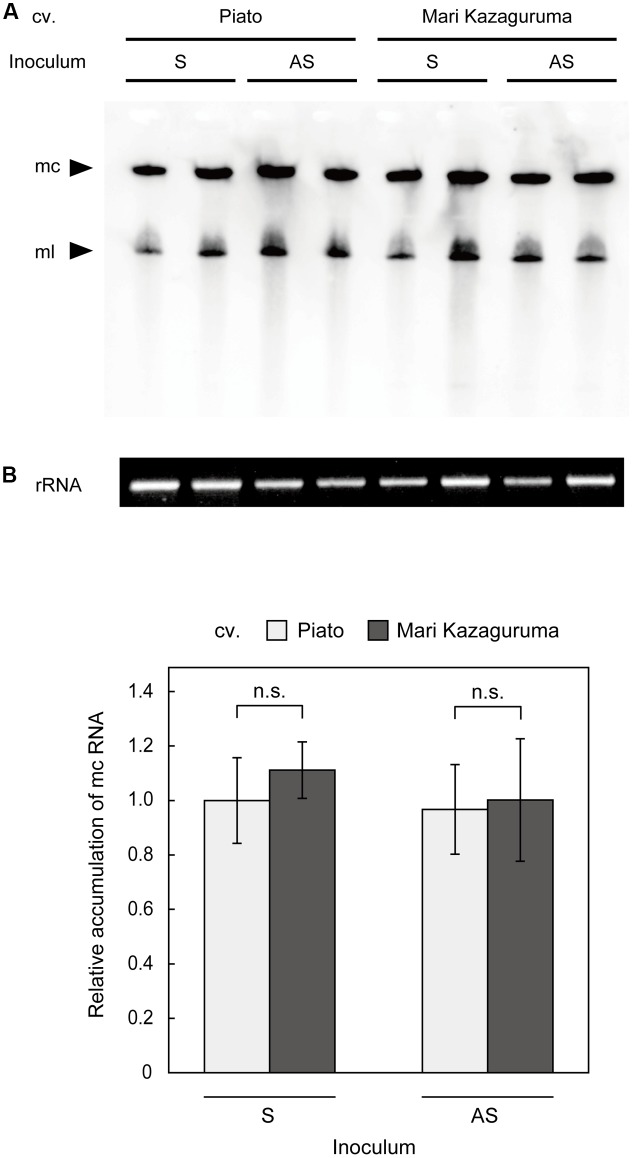
Detection of (+) CSVd in agro-infiltrated leaves of Piato and Mari Kazaguruma by northern blot. Total RNA extracted from pBIK201:CSVd-2S- and pBIK201:CSVd-2AS-inoculated leaves was separated on a 5% acrylamide gel containing 8 M urea and transferred to a nylon membrane. Hybridization was performed at 65°C using a DIG-labeled CSVd full-length probe. **(A)** CSVd detection in inoculated leaves at 7 dpi. CSVd signals were detected using an antisense CSVd probe. **(B)** Band intensities corresponding to CSVd mc forms were quantified using the gel analysis function of ImageJ software. The same RNA dilutions used for northern blotting were separated in parallel on formaldehyde-containing agarose gels and stained with ethidium bromide as shown at the bottom of **(A)**, and band intensities of 26S rRNA were measured to standardize concentrations of RNA samples. Data are represented as means of six biological replications and bars show standard errors. The vertical axis shows CSVd circular accumulation levels relative to pBIK201:CSVd-2S-inoculated leaves. Student’s *t*-test indicated no statistically significant difference between cultivars (*p* < 0.05). S, pBIK201:CSVd-2S-inoculated leaves; AS, pBIK201:CSVd-2AS-inoculated leaves; mc, monomeric circular CSVd; ml, monomeric linear CSVd.

To analyze viroid replication in plant cells, protoplast systems have been successfully applied in *Nicotiana* (Qi and Ding, 2002) and *Citrus* (Hajeri et al., 2011). However, a Piato protoplast system did not produce reliable results in our previous study, probably due to a low transfection ratio or slow CSVd replication relative to cell proliferation (unpublished). Moreover, creating protoplasts with equivalent qualities from two different cultivars is difficult. Thus, we decided to make comparative analysis of CSVd replication using agro-infiltrated leaves. Although this analysis cannot exclude the effects of CSVd movement within a leaf, CSVd accumulation levels in these leaves should approximate CSVd replication efficiencies in different cultivars. We examined the levels of CSVd accumulation in pBIK201:CSVd-2S- or pBIK201:CSVd-2AS-inoculated leaves of Piato and Mari Kazaguruma at 7 dpi. In both inoculated cultivars, two clear bands, corresponding to CSVd linear and circular RNAs, were detected by Northern-blot using antisense CSVd probes (**Figure 5A**). Quantitative analysis of accumulation levels of mc RNAs by ImageJ software indicated no statistically significant differences between the cultivars (**Figure 5B**). Interestingly, the signals were similar in both cultivars independently on whether the plants were inoculated with plasmids expressing the plus or the minus CSVd RNAs (pBIK201:CSVd-2S and pBIK201:CSVd-2AS), thus indicating a similar capacity of supporting viroid replication.

### CSVd Distribution in Inoculated Leaves

**FIGURE 6:**
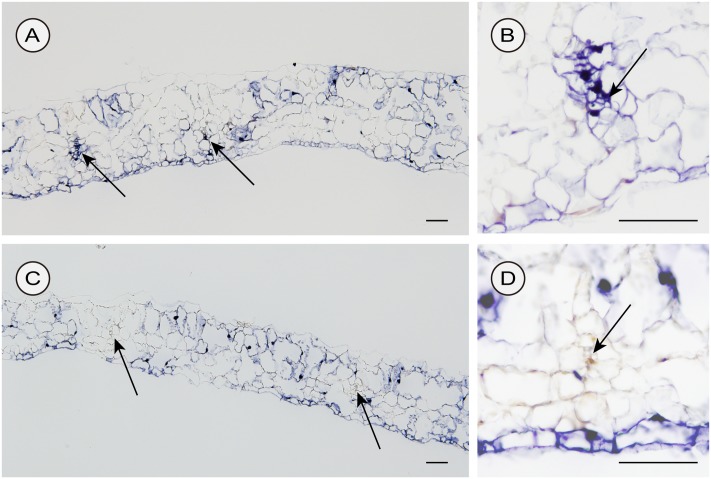
Detection of sense CSVd RNA in transversal sections of CSVd-inoculated leaves of Piato **(A,B)** and Mari Kazaguruma **(C,D)** by *in situ* hybridization. EHA105 harboring pBIK201:CSVd-2S was infiltrated into the fifth youngest expanded leaves. Tissues were fixed at 14 days post inoculation. DIG-labeled CSVd antisense RNA probe was used for hybridization. Arrows indicate phloem. Bar = 100 μm.

**FIGURE 7:**
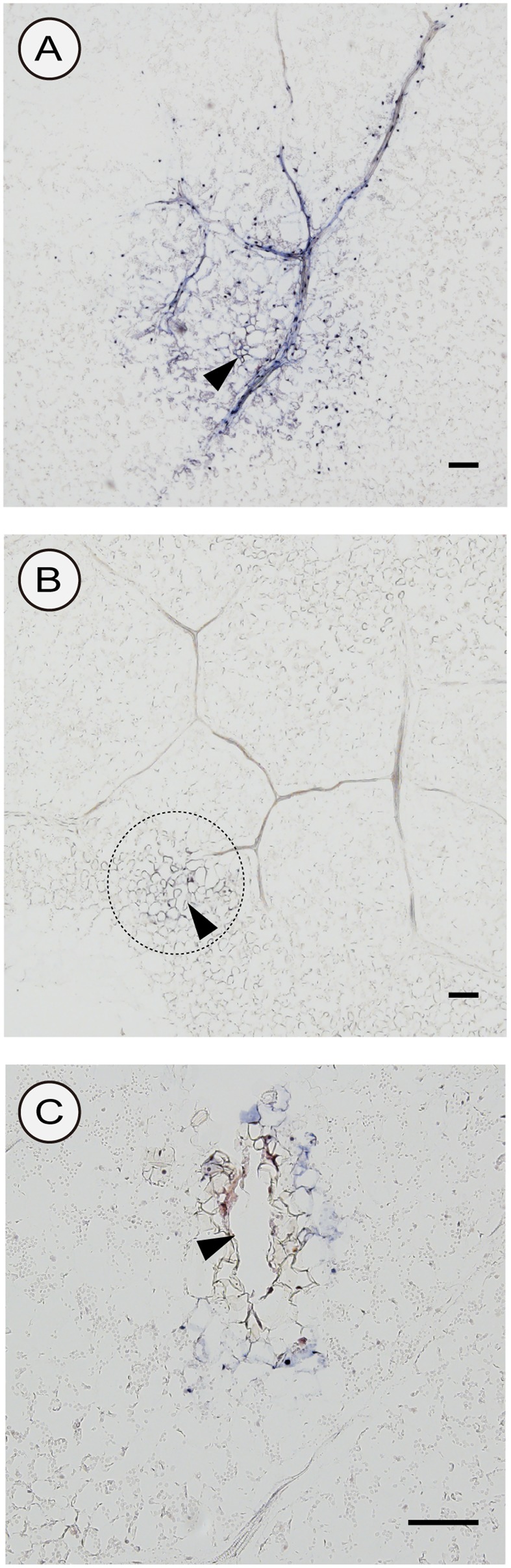
Detection of sense CSVd RNA in peridermal sections of CSVd-inoculated leaves of Piato **(A)** and Mari Kazaguruma **(B,C)** by *in situ* hybridization. A syringe needle was dipped in cultures of EHA105 harboring pBIK201:CSVd-2S and then used for piercing the fifth youngest expanded leaves. Tissues were fixed at 5 days after inoculation. DIG-labeled CSVd antisense RNA probe was used for hybridization. The dashed circle in **(B)** indicates the inoculated site. Arrowheads indicate CSVd-inoculated sites. Bar = 100 μm.

We used two different inoculation methods to examine CSVd distribution in lower leaves: the agroinfiltration method (with a syringe without a needle) and the agroinfection using a contaminated needle, which are expected to allow investigations on the CSVd distribution patterns when the infection initiates in many cells almost simultaneously (agroinfiltration) and in a restricted number of cells, respectively. In the two cultivars agroinfiltrated with pBIK201:CSVd-2S, CSVd signals were observed over a large part of transversal sections and were strongly concentrated in the nucleus of the cells. In Piato, phloem showed intense signals at 14 dpi (arrows; **Figures 6A,B**). In Mari Kazaguruma, sieve elements and surrounding parenchyma cells were CSVd-negative, even when their nearby spongy mesophyll cells displayed strong signals (arrows; **Figures 6C,D**). In this experiment, we used three Piato Mari Kazaguruma plants, and similar results were obtained from the other sections. The presence of a white patch of cells in both cultivars indicated that CSVd infection did not start from all cells at the same time in this experimental system (**Figures 6A,C**). And it was assumed that absence of CSVd in phloem of Mari Kazaguruma was related to the speed of CSVd distribution from the initially infected cells. This assumption was supported by observations on CSVd-inoculated plants by agroinfection with a needle. In this experiment, we analyzed paradermal sections obtained from four Piato and three Mari Kazaguruma plants and representative images were shown in **Figure 7**. In Piato, CSVd RNA was detectable as a patch of blue-stained cells around the injured site at 5 dpi (**Figure 7A**). These signals were strongly concentrated in the nucleus of many infected cells, and strong signals were observed even in cells which were >20 cells distant from the inoculated site. In these sections, signals were spreading from the injured site to distant cells along the vasculature. In the vasculature, CSVd localized in sieve elements, accompanied by signals in the nucleus of companion cells and bundle sheath cells (**Figure 7A**). In sections of Mari Kazaguruma inoculated leaves, CSVd could be detected by nuclear-localized signals around the injured sites; in contrast, infected cells were relatively fewer than in Piato (**Figures 7B,C**). It was suggested that CSVd cell-to-cell movement in Mari Kazaguruma was slower than that in Piato.

## Discussion

In this study, we compared systemic translocation, local accumulation, and intra-leaf movement of CSVd in two chrysanthemum cultivars: susceptible cultivar Piato and resistant cultivar Mari Kazaguruma. Viroids use phloem for long-distance translocation from source leaves to sink leaves (Palukaitis, 1987; Zhu et al., 2001). CSVd was expected to be supplied from infected rootstocks or agro-infiltrated leaves to young leaves, which are strong sink tissues in tested plants. Our grafting experiments showed that when Mari Kazaguruma plants were grafted onto P4, CSVd was detectable in the uppermost leaves, but after removal of P4 rootstocks, CSVd detection ratio and CSVd titers drastically decreased (**Figure 2**). Furthermore, in agro-infiltrated Mari Kazaguruma, CSVd systemic translocation was not observed within 25 days. The results of tissue blotting suggested that CSVd was arrested in an inoculated leaf of Mari Kazaguruma (**Figure 4A**), however, inoculated leaves of Piato and Mari Kazaguruma accumulated mature CSVd RNA at the equivalent levels after agro-infiltration (**Figure 5**). A 20-day infection duration was sufficient for CSVd to start up long-distance translocation in Piato (**Table 1** and **Figure 4A**). In fact, CSVd was detectable in phloem in agro-infiltrated leaves at 14 dpi (**Figure 6A**). However, CSVd was absent in phloem of Mari Kazaguruma at the same time (**Figure 6C**). Furthermore, CSVd spread from initial infection sites relatively slowly in Mari Kazaguruma compared to Piato (**Figure 7**). These results suggested that in Mari Kazaguruma, ineffectiveness in cell-to-cell movement of CSVd resulted in infrequent entry to the vasculature in leaves, resulting in poor systemic infection. Although CSVd cell-to-cell movement was not perfectly blocked in Mari Kazaguruma, its speed was slow enough to impede acropetal CSVd movement, as shown in our grafting experiments (**Figure 3**). In the present study, however, we could not determine the mechanism(s) of how CSVd movement was inhibited, and we could not clearly determine where the inhibitory event(s) occurred.

Ding et al. (1997) showed that PSTVd moves cell-to-cell through plasmodesmata (PD). Although direct evidences have not been reported yet, viroids in the *Avsunviroidae* are also thought to move cell-to-cell through PD (Ding, 2009). PD are channels which symplastically connect cells, and plant viruses and viroids move cell-to-cell through PD (Roberts and Oparka, 2003; Lucas et al., 2009). It is known that PD have a size exclusion limit, but many viruses encode proteins that can broaden the size exclusion limit and enable their cell-to-cell movement (Lucas, 2006). Deposition of callose in PD is known to limit the size of PD (Tilsner et al., 2016) and has a role in inhibiting pathogen distribution (De Storme and Geelen, 2014; Ellinger and Voigt, 2014). Recently, callose deposition was shown to inhibit viroid distribution (Zhang et al., 2014; Adkar-Purushothama et al., 2015). In our grafting experiment, Mari Kazaguruma systemic leaves accumulated CChMVd much more than CSVd (**Figure 3**). Although the possibility of Mari Kazaguruma having an extremely high capacity for CChMVd replication could not be excluded, the scenario is unlikely in which the number or size of PD in Mari Kazaguruma are too small for viroid distribution. Rather, this observation suggests that CSVd sequence-specific events are involved. Evidence suggests that translocation of PSTVd across a specific cellular boundary, such as bundle sheath to mesophyll (Qi et al., 2004), vascular entry (Zhong et al., 2007), or palisade mesophyll to spongy mesophyll (Takeda et al., 2011), requires RNA motifs with specific tertiary structures. In these papers, the authors proposed the existence of endogenous machinery that directly recognizes viroid tertiary structures. In our study, CSVd distribution seemed to be restricted at the mesophyll to bundle sheath boundary in agro-infiltrated leaves at 14 dpi (**Figure 6D**). However, as seen in **Figure 7**, it was likely that CSVd distribution in Mari Kazaguruma was delayed in spongy mesophyll cells also. It remains unknown whether there is a specific cellular boundary which restricts CSVd spread in Mari Kazaguruma or is it that all cells in Mari Kazaguruma possess inhibitory effects for CSVd spread. Host factors that facilitate viroid cell-to-cell movement through PD have not been identified. However, it was previously shown that viroid-binding protein 1 (bromodomain-containing host protein 1) in *Nicotiana* and tomato (Martínez de Alba et al., 2003; Kalantidis et al., 2007; Chaturvedi et al., 2014) and phloem lectin PP2 in cucumber (Owens et al., 2001; Gómez and Pallás, 2004) interact with viroid RNA and have roles in nuclear importation and long-distance translocation, respectively. Maniataki et al. (2003) suggested that the low affinity for viroid-binding protein 1 was responsible for poor infectivity of *Hop stunt viroid* in tomato. When viroids are moving from a cell to an another cell, the process includes import/export steps between cytosol and organelles where replication events take place. The different localization pattern of CSVd and CChMVd, with the former replicating and accumulating in nuclei and the latter in plastids, should be considered to find a possible explanation to the different susceptibility of the two cultivars to these two viroids. However, data reported here are too preliminary to address this question. As a possible explanation for different susceptibility to CSVd infection in two cultivars, we propose that expression levels, sites, or affinity toward CSVd RNA of host factors, which are involved in CSVd translocation, differ between these two cultivars.

RNA silencing is another possible explanation for CSVd resistance in Mari Kazaguruma. RNA silencing provides defense systems which protect plants from invasion by exogenous RNA replicons such as virus and viroids (Baulcombe, 2004; Voinnet, 2005). Infection of viroids induces accumulation of small interfering RNA (siRNA) in host plants (Itaya et al., 2001; Martínez de Alba et al., 2002; Markarian et al., 2004). Sano and Matsuura (2004) suggested that recovery from severe symptoms in PSTVd-infected tomato was induced by sequence-specific RNA degradation. The RNA interference pathway was successfully applied to produce viroid-resistant tomato (Schwind et al., 2009), *Nicotiana* (Kasai et al., 2013), and chrysanthemum (Jo et al., 2015) through transgenic approaches. Di Serio et al. (2010) showed that RNA silencing is involved in preventing PSTVd invasion of the shoot apex in *Nicotiana*. In our previous study, we showed that CSVd absence in shoot apices of CSVd-inoculated plants were a common characteristic in resistant chrysanthemum cultivars (Nabeshima et al., 2012). Hirai et al. (2008) showed that antiviral RNA silencing activity against *Tomato mosaic virus* in tobacco (*Nicotiana tabacum*) leaves results in the development of mosaic-patterned *Tomato mosaic virus* distribution and mosaic symptoms. Thus, in response to exogenous RNA pathogen invention, RNA silencing machinery can act to establish new boundary regions. It may be possible that Mari Kazaguruma possesses a strong RNA silencing activity to prevent CSVd distribution. In our preliminary experiments, we detected CSVd siRNA from infected Piato, but not in CSVd-inoculated Mari Kazaguruma (data not shown). However, the CSVd/*Actin* ratio in Mari Kazaguruma plants was 1.0 × 10^-3^ less than that in Piato plants at that time, making it hard to detect CSVd degradation products. We previously established an *Agrobacterium*-mediated inoculation method for a chrysanthemum-CSVd system (Nabeshima et al., 2016). In this study, it was possible to make CSVd titers higher in Mari Kazaguruma tissues using the MAS promoter. This system will enable us to investigate CSVd degradation activity in resistant cultivars, and future work should focus on this issue.

In *Arabidopsis thaliana*, no viroid has been reported to be infectious so far. Daròs and Flores (2004) fed sense or antisense dimeric transcripts of representative viroids in *A. thaliana* using an exogenous promoter. They found that members of the *Pospiviroidae* could replicate in *A. thaliana* but the efficiency was low and systemic spread did not occur (Daròs and Flores, 2004). As for the Mari Kazaguruma-CSVd system, the combination displayed “low compatibility”; without exogenous CSVd feeding, Mari Kazaguruma kept CSVd titers very low but its replication and movement were not perfectly blocked. Among viroid studies, this type of combination is not well studied. In wild potato, two *Solanum berthaultii* clones were identified to have resistance to mechanical sap inoculation with PSTVd, but they did not show strong resistance to grafting-mediated inoculation (Singh, 1985). Those situations resemble the Mari Kazaguruma-CSVd system; however, their resistance mechanisms have not been elucidated yet. To date, several studies have focused on introducing viroid resistance by transgenic approaches (Kovalskaya and Hammond, 2014; Dalakouras et al., 2015; Flores et al., 2017), but non-transgenic approaches are elusive. In chrysanthemum, there are multiple resistance sources in cultivated species (Omori et al., 2009; Matsushita et al., 2012; Nabeshima et al., 2012, 2014). They are useful materials for commercial cultivation and breeding. From an agricultural view, it is important to elucidate and classify these resistances. In addition, they are also interesting materials for studying mechanisms that quantitatively regulate the viroid life cycle.

## Author Contributions

TN contributed to the conception or design of the work, collect data, data analysis, interpretation of data and writing of manuscript; MD contributed to data analysis, interpretation of data and final approval of the version; MH contributed to the conception or design of the work, data analysis, interpretation of data and final approval of the version.

## Conflict of Interest Statement

The authors declare that the research was conducted in the absence of any commercial or financial relationships that could be construed as a potential conflict of interest.
